# Importance of Imaging in Congenital Unilateral Vocal Fold Paralysis: A Case of Neck Neuroblastoma Presenting with Unilateral Vocal Fold Paralysis

**DOI:** 10.1155/2021/7368567

**Published:** 2021-12-06

**Authors:** W. X. Yeo, C. Y. Chan, K. K. H. Tan

**Affiliations:** Department of Otolaryngology, KK Women's and Children's Hospital, Singapore

## Abstract

Congenital vocal fold paralysis (VFP) is an important cause of respiratory compromise in infants. It can either be unilateral or bilateral, while imaging is routinely performed for bilateral VFP to evaluate for potential neurological causes, and such a practice may not be routine for unilateral VFP. While many of the unilateral VFP cases are idiopathic, the cause may occasionally be more sinister in nature, such as tumors. Therefore, unless an obvious cause of unilateral VFP is present (such as cardiac surgery or birth trauma), routine imaging of the brain, neck, and mediastinum should be performed for congenital unilateral VFP. We describe a rare case of a cervical neuroblastoma presenting with unilateral VFP that was detected only on imaging, thus highlighting its value and importance.

## 1. Introduction

Congenital vocal fold paralysis (VFP) is an important cause of respiratory compromise in infants [1]. It can either be bilateral or unilateral, with reported incidence of about 50% each [2]. Bilateral VFP often presents with severe airway obstruction such as stridor or respiratory distress, with up to 73% requiring tracheostomy [3]. Unilateral VFP, on the other hand, usually presents with abnormal cry or feeding difficulty, in addition to stridor [2]. Common causes of bilateral VFP include idiopathic, neurologic (such as Arnold-Chiari malformation), and birth trauma (especially with forceps delivery) [2]. Common causes of unilateral VFP include iatrogenic (injury to recurrent laryngeal nerve (RLN) or vagus nerve during cardiothoracic or neck surgeries), idiopathic, and birth trauma [2, 4]. Rarely, neoplasms involving the skull base, neck, or mediastinum—along the course of vagus nerve or RLN—can result in unilateral VFP [4]. In this report, we describe a case of a cervical neuroblastoma presenting with unilateral VFP that was detected only on imaging. The tumor was eventually excised, and adjuvant chemotherapy was administered.

## 2. Case Presentation

A full-term 30-day-old girl born via normal vaginal delivery with no significant perinatal history developed increasing dyspnea, hoarseness, cyanosis with feeding, and lethargy. She was treated for respiratory syncytial virus (RSV) pneumonia and required supplemental oxygen and parenteral feeding. The feeding with cyanosis persisted after five days, prompting a visit to our hospital for a second opinion. Upon arrival, the patient had an oxygen saturation of 89% on room air and was admitted to the ward for further management. The oxygen saturation improved to 100% with supplemental oxygen. She was alert with no audible stridor at rest but had mild subcostal retractions. The heart and breath sounds were normal. Otolaryngology was consulted and bedside nasoendoscopy reviewed a left vocal fold paralysis. The right vocal fold was mobile and the glottic gap was three millimeters. The remainder of the scope findings was normal. There were no palpable neck masses, and thorough examination of the ears and oral cavity was normal as well. Chest X-ray showed mild air space consolidation at the right perihilar region and possibly the left retrocardiac region, likely related to underlying pneumonia. The patient was treated presumably for aspiration pneumonia and was weaned off supplemental oxygen after a few days.

**Figure 1:**
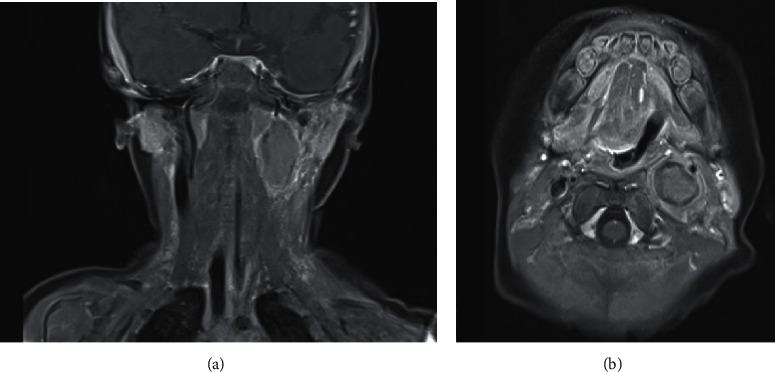
MRI T1-weighted images with fat saturation and contrast, coronal (a) and axial (b) views, depicting a non-enhancing left carotid space tumor. Neuroblastomas typically show variable and heterogeneous enhancement and are of relatively low signal intensity on T1-weighted images and high signal intensity on T2-weighted images [5].

To evaluate the unilateral vocal fold paralysis, a magnetic resonance imaging (MRI) scan was performed. This revealed a nonenhancing left carotid space mass measuring 2.4 × 1.5 × 1.3 cm (Figure 1), with compression on the left internal jugular vein and displacement of the internal and external carotid arteries anteriorly. Ultrasound scan of the lesion showed multiple intralesional calcifications, a common finding seen in neuroblastoma [5, 6]. At this point, the main differential diagnosis was a cervical neuroblastoma; other differential diagnoses include nerve sheath tumors such as schwannoma or neurofibroma.

The patient underwent excision of the left neck mass. Intraoperatively, the mass was seen arising from within the carotid sheath and extending superiorly into the base of the skull. Attempts were made to completely excise the mass, but this was not possible due to tumor adherence to the skull base. The mass also encased the left vagus nerve and was adherent to the left hypoglossal nerve; it was dissected free from these nerves which were identified and preserved. A few prominent adjacent jugulodigastric nodes were also excised. Final histology of the mass returned as poorly-differentiated neuroblastoma, with metastatic tumor present in two of the three adjacent jugulodigastric nodes.

Postoperatively, the patient underwent MRI of the neck, chest, and abdomen. This showed residual tumor at the skull base, a few prominent right cervical lymph nodes (likely reactive), and no metastatic disease elsewhere in the body, especially in the mediastinum, retroperitoneum, and adrenal glands. Aside from persistent dysphagia requiring nasogastric tube (NGT) feeding, the patient had an uneventful recovery and was discharged well on postoperative day seven.

The multidisciplinary tumor board assessed the tumor to be of low-risk and recommended close observation, as neuroblastomas may spontaneously regress [7]. Unfortunately, follow-up MRI a month later showed disease progression. There was tumor extension into the left parapharyngeal space and further extension superiorly into the skull base. Bilateral multiple enlarged enhancing cervical lymphadenopathy was also present, suggestive of metastasis. The tumor was staged as Stage 2B (International Neuroblastoma Staging System), and the patient was commenced on chemotherapy in view of progressive disease. The patient completed three cycles of OJEC, vincristine [O], carboplatin [J], etoposide [E], cyclophosphamide [C], and one cycle of OJE by six months of age. Post-chemotherapy scans showed a reduction in the size of the tumor. One year after treatment, the tumor has remained stable in size, with plans for further observation with serial imaging.

Clinically, the patient's hoarseness improved over time, and she was initiated on oral feeding by her parents at eight months of age, who declined formal swallowing assessment before initiation. The patient tolerated this well with no clinical signs of aspiration. At age one year, she was developmentally normal and could babble, cruise, and wave on command. Nasoendoscopic examination at age 15 months showed a persistent left vocal fold palsy with right vocal fold compensation. The glottic gap remained adequate.

## 3. Discussion

The approach to VFP involves eliciting a proper history and performing a thorough clinical examination for evidence of congenital or neurological anomalies, iatrogenic surgical causes, or birth trauma [8]. This is followed by visualization of the vocal folds via flexible or direct laryngoscopy, with the latter allowing for palpation to differentiate vocal fold paralysis from fixation [2]. Alternatively, laryngeal electromyography (EMG) has been found to be useful in differentiating fixation from paralysis and in assisting with prognosis [9]. Laryngeal ultrasonography is another useful adjunct that can be employed to aid in the diagnosis of VFP [10], especially with poor visualization on laryngoscopy due to collapsing supraglottic tissues commonly seen in infants.

Once the diagnosis of VFP is established, its underlying cause needs to be investigated. However, bilateral VFP cases routinely undergo imaging to evaluate for potential neurological causes [11, 12], such a practice may not be routine for unilateral VFP. While the cause of unilateral VFP is often idiopathic [2, 4], this case report illustrates the importance of imaging to evaluate for other causes of unilateral VFP, especially in the absence of an obvious cause (e.g., recent cardiothoracic surgery). In fact, neurological causes such as Arnold-Chiari malformation have been reported to cause unilateral VFP as well [13], even though it is more commonly implicated in bilateral VFP. There have also been reports of thyroid carcinoma in newborn [14] and pediatric neurological malignancies resulting in both unilateral and bilateral VFP [15]. It is therefore not inconceivable that pathologies anywhere along the course of the vagus nerve from skull base to the RLN can present as congenital unilateral VFP, as evident in this case report.

In this case, cervical neuroblastoma was the cause of the patient's congenital unilateral VFP. Neuroblastomas are the most common extracerebral solid tumors of infancy and childhood, with a reported incidence of 10.2 cases per million children under 15 years of age [16]. They arise from neural crest cells and may develop wherever sympathetic nervous tissue is found [17]. The most common sites of origin are in the adrenal medulla and the retroperitoneum, while about three percent of neuroblastomas develop in the head and neck region [18]. A peculiar feature of neuroblastoma is its tendency to show spontaneous regression, especially in infants with localized neuroblastoma [19]. Treatment modalities include surgery, chemotherapy, and/or radiotherapy, and management is dependent on the stage, site, and risk stratification of the disease [16].

In conclusion, the authors propose that unless an obvious cause of unilateral VFP is present (such as cardiac surgery or birth trauma), routine imaging of the brain, neck, and mediastinum should be performed to investigate congenital unilateral VFP.

## Data Availability

Data are not available due to patient privacy and prevailing privacy laws.
